# Social Determinants of Health, Blood Pressure Classification, and Incident Stroke Among Chinese Adults

**DOI:** 10.1001/jamanetworkopen.2024.51844

**Published:** 2024-12-23

**Authors:** Yanchen Zhu, Shiping Wu, Weida Qiu, Jiabin Wang, Yingqing Feng, Chaolei Chen

**Affiliations:** 1 Department of Cardiology, Guangdong Cardiovascular Institute, Guangdong Provincial People’s Hospital (Guangdong Academy of Medical Sciences), Southern Medical University, Guangzhou, China; 2 Global Health Research Center, Guangdong Provincial People’s Hospital (Guangdong Academy of Medical Sciences), Southern Medical University, Guangzhou, China; 3 Hypertension Laboratory, Cardiovascular Disease Center of Guangdong Province, Guangdong Cardiovascular Institute, Guangdong Provincial People’s Hospital (Guangdong Academy of Medical Sciences), Southern Medical University, Guangzhou, China

## Abstract

**Importance:**

Although cumulative evidence suggests that higher blood pressure (BP) and a greater burden of social determinants of health (SDOH) are associated with an increased risk of stroke, few studies have examined whether SDOH burden modifies the association between BP and stroke risk.

**Objective:**

To evaluate whether the association between BP classification and stroke risk differs by SDOH burden among Chinese adults.

**Design, Setting, and Participants:**

In this cohort study, analyses were conducted among 90 850 participants in the prospective subcohort of the China Patient-Centered Evaluative Assessment of Cardiac Events Million Persons Project, with recruitment from January 1, 2016, to December 31, 2020. Participants without cardiovascular diseases and with complete data were included and followed up for stroke events until June 30, 2023.

**Exposures:**

Social determinants of health burden was defined using 5 components: educational attainment, economic stability, health care access, social support, and urban vs rural residence. Blood pressure was categorized according to the 2017 American College of Cardiology/American Heart Association BP guideline.

**Main Outcomes and Measures:**

Multivariable Cox proportional hazards regression was used to estimate hazard ratios (HRs) and 95% CIs for associations of BP classification with incident stroke stratified by SDOH burden groups.

**Results:**

The study included 90 850 participants (median age, 54.0 years [IQR, 46.0-62.0 years]; 55 390 women [61.0%]). During a median follow-up of 5.0 years (IQR, 4.1-5.8 years), 4408 incident stroke events were recorded. Compared with normal BP, elevated BP (HR, 1.33 [95% CI, 1.17-1.52]), stage 1 hypertension (HR, 1.60 [95% CI, 1.43-1.78]), and stage 2 hypertension (HR, 1.79 [95% CI, 1.61-2.00]) were associated with stroke among participants with high SDOH burden, while only stage 2 hypertension (HR, 1.52 [95% CI, 1.20-1.93]) was associated with stroke among those with low SDOH burden, with a significant multiplicative interaction of SDOH burden and BP classification (*P* = .03). Participants with high SDOH burden and stage 2 hypertension had the highest risk of stroke compared with participants with low SDOH burden and normal BP (HR, 2.13 [95% CI, 1.75-2.60]).

**Conclusions and Relevance:**

This study suggests that higher BP levels, even at lower stages of hypertension, pose a greater stroke risk for individuals with high SDOH burden. Early surveillance and intervention for high BP should be emphasized in socioeconomically disadvantaged populations.

## Key Points

**Question:**

Does the association between blood pressure (BP) classification, according to the 2017 American College of Cardiology/American Heart Association guideline, and incident stroke differ by burden of social determinants of health (SDOH)?

**Findings:**

In this cohort study of 90 850 Chinese adults, elevated BP, stage 1 hypertension, and stage 2 hypertension were associated with incident stroke among those with high SDOH burden. Only stage 2 hypertension was associated with incident stroke among those with low SDOH burden.

**Meaning:**

This study suggests that higher BP levels, even at lower hypertension stages, are associated with stroke risk for individuals with high SDOH burden, emphasizing the need for targeted interventions in vulnerable populations.

## Teaser

This cohort study evaluates whether the association between blood pressure classification and stroke risk differs by burden of social determinants of health (SDOH) among Chinese adults.

## Introduction

Stroke is the world’s second leading cause of death and third leading cause of disability, and its burden is rapidly increasing in low-income and middle-income countries.^1,2^ Therefore, understanding and controlling the factors that could lead to stroke are crucial. Evidence has shown that hypertension is one of the most common and modifiable risk factors for stroke,^3^ accounting for 65.1% of the disability-adjusted life-years lost due to stroke in low-income and middle-income countries and 59.8% in high-income countries.^4^ The long-term effects of hypertension, such as inducing smooth muscle hypertrophy, reducing vascular compliance, and increasing shear stress,^5^ can be independently associated with the risk of ischemic and intracerebral hemorrhagic strokes.^6^ According to the scientific literature, more than 50% of ischemic strokes and 70% of hemorrhagic strokes occur among individuals with hypertension.^7,8,9^ Therefore, appropriate blood pressure (BP) control is an essential measure in reducing the risk of stroke.

In studies conducted in Western populations, many researchers have identified a significant correlation between the burden of unfavorable social determinants of health (SDOH) and the risk of stroke.^10,11^ In a prospective cohort study conducted in the US, researchers further proposed that SDOH have an accumulative association with the risk of stroke, wherein as the number of SDOH increases, so does the risk of stroke.^12^ However, the mechanisms underlying the association between SDOH and stroke are complex and unclear. Current theories suggest that the association between SDOH and stroke may result largely from indirect pathways involving cardiovascular factors.^13,14^ Interventions targeting these factors could have a positive association with the prevention and management of stroke in groups with different burdens of SDOH.^12^ Currently, high SDOH burden is commonly observed among adults with hypertension and is associated with BP control.^15^ Moreover, controlling BP has long been considered as an essential means of preventing stroke.^16^ Given these aspects, it is crucial to investigate the association between SDOH, BP, and stroke, and to examine whether the association between BP and stroke varies across different levels of SDOH burden.

However, research integrating SDOH-related factors with BP and cardiovascular disease is currently limited and primarily focused on White populations.^17,18,19^ The association between SDOH, BP, and stroke risk among the Chinese population is currently unclear. Moreover, although the European Stroke Organization has recommended different BP management methods based on stroke subtypes and cause,^20^ the BP target is unknown for individuals with high SDOH burden. Therefore, this study aims to investigate whether the association between BP classification, recommended by the 2017 American College of Cardiology/American Heart Association (ACC/AHA) BP guideline,^21^ and risk of stroke differs by SDOH burden among Chinese adults.

## Methods

### Study Design and Participants

The present cohort study was based on a subcohort of the China Patient-Centered Evaluative Assessment of Cardiac Events Million Persons Project that recruited 102 358 community residents from 8 locations in Guangdong province from January 1, 2016, to December 31, 2020, with follow-up until June 30, 2023. The information about this project has been comprehensively documented in previous literature.^22,23^ This project is a nationwide, government-funded, community-based study focusing on the burden of cardiovascular diseases in China. In the current study, men and women aged 35 to 75 years were included. We excluded 6255 individuals with missing data for any component of SDOH, 2725 individuals who had prevalent stroke at baseline, and 1993 individuals who had prevalent coronary heart disease or heart failure at baseline. Finally, 535 individuals who had no follow-up data were excluded. Ultimately, we included 90 850 participants for the final analysis (eFigure 1 in Supplement 1). We compared the baseline characteristics of the totally excluded population and the population excluded due to missing SDOH data with those of the included population and found no significant differences (eTable 1 in Supplement 1). This study was approved by the Central Ethics Committee at the China National Center for Cardiovascular Disease and the Ethics Committee of Guangdong Provincial People’s Hospital. All participants provided written informed consent before their involvement in the study. This study followed the Strengthening the Reporting of Observational Studies in Epidemiology (STROBE) reporting guideline.

### Data Collection and Variables

During interviews conducted by trained staff members, we recorded data on the exposure variables, including SDOH factors and BP readings, and covariates, including age, sex (male or female), smoking status (current smokers or not), drinking status (current drinker or not), body mass index (BMI), diabetes, dyslipidemia, and use of antihypertensive medications. We used standard procedures to measure height and body weight and then calculated BMI by dividing the weight in kilograms by the square of the height in meters. Blood pressure was measured twice on the right upper arm after 5 minutes of rest in a seated position using an electronic BP monitor (HEM-7430; Omron), and the mean value was used. Fasting plasma glucose (BeneCheck BK6-20M Multi-Monitoring System; Suzhou Pu Chun Tang Biotechnology) and lipid profiles (CardioChek PA Analyzer; Polymer Technology Systems) were obtained from blood samples.

### Definition of SDOH Burden

Social determinants of health are societal factors that consist of structural elements within society and the conditions of everyday life; they are significantly associated with health inequalities across and within countries.^24^ In this study, we developed a comprehensive SDOH framework based on 5 individual components across 2 domains, including individual-level and area-level SDOH. The individual-level SDOH included educational attainment, economic stability, and health care access, and the area-level SDOH included social support and urban vs rural residence.^13,25,26,27^ All information was collected based on the participants’ self-reported responses via a structured questionnaire. Each component was classified as unfavorable or favorable, with a value of 0 assigned to the former and 1 to the latter, with a maximum score of 5. To calculate an aggregate SDOH score, we added the scores for individual components. Consequently, a lower aggregate SDOH score indicated higher SDOH burden. In line with a previous work, an overall SDOH score of 0 to 3 indicated a high SDOH burden, while an overall score of 4 or 5 indicated a low SDOH burden.^28^ Detailed definitions of SDOH and scoring criteria are shown in eTable 2 in Supplement 1.

### Definition of BP Classification

According to the 2017 ACC/AHA BP guideline,^21^ we categorized participants based on BP readings as having normal BP (systolic BP [SBP] <120 mm Hg and diastolic BP [DBP] <80 mm Hg), elevated BP (SBP of 120-129 mm Hg and DBP <80 mm Hg), stage 1 hypertension (SBP of 130-139 mm Hg or DBP of 80-89 mm Hg), and stage 2 hypertension (SBP ≥140 mm Hg or DBP ≥90 mm Hg).^29^

### Study Outcome

The outcome of interest was incident stroke, defined as hospitalization for stroke or death due to stroke. Researchers conducted an annual assessment of stroke incidence during the follow-up period. Hospitalization information was extracted from the hospital episode records, and death events were extracted from the Chinese Center for Disease Control and Prevention National Mortality Surveillance System and Vital Registration, where stroke cases were determined using the *International Statistical Classification of Diseases and Related Health Problems, Tenth Revision*, codes for stroke (I60-I64).^28,30^ All outcome events were determined by linking national identity card numbers and were adjudicated by study investigators blinded to the participants’ SDOH burden and BP status. The follow-up period was calculated from the enrollment date (baseline) to the occurrence of incident stroke, nonstroke death, loss to follow-up, or the end of follow-up (June 30, 2023), whichever occurred first.

### Statistical Analysis

There were no missing values for any covariates in this study, so no imputation or other methods for handling missing data were performed. Baseline characteristics were compared according to SDOH groups and BP classifications. The distribution of continuous variables was examined using the Kolmogorov-Smirnov test and was presented as median (IQR) values if not normally distributed. Categorical variables were presented as numbers and percentages and were compared using the χ^2^ test.

Individual associations of SDOH burden and BP classification with incident stroke were assessed using Cox proportional hazards regression models. Model 1 was adjusted for age and sex. Model 2 was additionally adjusted for smoking status, drinking status, BMI, dyslipidemia, diabetes, and antihypertensive medications. Model 3 was further adjusted for SDOH burden (for the analysis of BP classification) and BP classification (for the analysis of SDOH burden). We investigated the variations of associations between BP classification and incident stroke within SDOH burden groups using Cox proportional hazards regression models. To investigate the multiplicative interaction of SDOH burden and BP classification, we introduced a cross-product term into the Cox proportional hazards regression model; the multiplicative interactive effect was determined if *P* < .05 for the interaction term. We applied restricted cubic spline regression models conducted with 3 knots at the 25th, 50th, and 75th percentiles of systolic and diastolic BP to explore the potential nonlinear and dose-response associations between SBP and DBP and stroke within SDOH burden groups. Furthermore, we explored the joint associations of SDOH burden and BP classification with incident stroke by using participants with low SDOH burden and normal BP as the reference group.

Considering the potential associations of the use of antihypertensive medications with stroke outcomes, we conducted stratified analyses and repeated analyses according to use of antihypertensive medications at baseline (yes vs no). In addition, we conducted several sensitivity analyses to test the robustness of the results. First, we excluded participants who had experienced a stroke within 1 year of follow-up to minimize the potential for reverse causality. Second, we used the BP classification according to the 2023 European Society of Hypertension (ESH) BP guideline to determine the consistency of the results.^31^

All statistical analyses were performed using R, version 4.4.1 (R Project for Statistical Computing). All tests were 2-tailed, and *P* < .05 was considered statistically significant.

## Results

### Population Characteristics

The analytic sample included 90 850 participants (median age, 54.0 years [46.0-62.0 years]; 55 390 women [61.0%] and 35 460 men [39.0%]). Detailed participant demographics according to SDOH burden (20 137 participants [22.2%] with low SDOH burden and 70 713 participants [77.8%] with high SDOH burden) are shown in Table 1. Adults with a high SDOH burden were older and more likely to be women, using antihypertensive medications, and have diabetes. Detailed participant demographics according to the BP classification (normal BP, 26 834 [29.5%]; elevated BP, 11 772 [13.0%]; stage 1 hypertension, 24 560 [27.0%]; and stage 2 hypertension, 27 684 [30.5%]) are in eTable 3 in Supplement 1.

**Table 1.** Baseline Characteristics of Participants According to SDOH Burden

Characteristic	Total population (N = 90 850)	Low burden of SDOH (n = 20 137)	High burden of SDOH (n = 70 713)	*P* value
Age, median (IQR), y	54.0 (46.0-62.0)	51.0 (44.0-60.0)	54.0 (46.0-62.0)	<.001
Sex, No. (%)				
Male	35 460 (39.0)	8643 (42.9)	26 817 (37.9)	<.001
Female	55 390 (61.0)	11 494 (57.1)	43 896 (62.1)	
Educational attainment, No. (%)				
Less than high school	87 927 (96.8)	17 518 (87.0)	70 409 (99.6)	<.001
High school or above	2923 (3.2)	2619 (13.0)	304 (0.4)	
Household income, No. (%)				
<¥50 000^a^	47 191 (51.9)	517 (2.6)	46 674 (66.0)	<.001
≥¥50 000	43 659 (48.1)	19 620 (97.4)	24 039 (34.0)	
Health insurance, No. (%)				
Uninsured	3964 (4.4)	72 (0.4)	3892 (5.5)	<.001
Insured	86 886 (95.6)	20 065 (99.6)	66 821 (94.5)	
Social support, No. (%)				
Unmarried	5004 (5.5)	44 (0.2)	4960 (7.0)	<.001
Married	85 846 (94.5)	20 093 (99.8)	65 753 (93.0)	
Urban vs rural residence, No. (%)				
Rural	48 187 (53.0)	754 (3.7)	47 433 (67.1)	<.001
Urban	42 663 (47.0)	19 383 (96.3)	23 280 (32.9)	
BP classification, No. (%)				
Normal BP	26 834 (29.5)	6652 (33.0)	20 182 (28.5)	<.001
Elevated BP	11 772 (13.0)	2390 (11.9)	9382 (13.3)	
Stage 1 hypertension	24 560 (27.0)	5477 (27.2)	19 083 (27.0)	
Stage 2 hypertension	27 684 (30.5)	5618 (27.9)	22 066 (31.2)	
Antihypertensive medications, No. (%)	16 105 (17.7)	3191 (15.8)	12 914 (18.3)	<.001
BMI, median (IQR)	23.9 (21.8-26.1)	23.9 (21.8-26.1)	23.9 (21.8-26.2)	.22
Current smoker, No. (%)	15 703 (17.3)	3573 (17.7)	12 130 (17.2)	.052
Current drinker, No. (%)	4811 (5.3)	1175 (5.8)	3636 (5.1)	<.001
Diabetes, No. (%)	13 985 (15.4)	2569 (12.8)	11 416 (16.1)	<.001
Dyslipidemia, No. (%)	16 462 (18.1)	3847 (19.1)	12 615 (17.8)	<.001

Abbreviations: BMI, body mass index (calculated as weight in kilograms divided by height in meters squared); BP, blood pressure; SDOH, social determinants of health.

^a^ ¥50 000 was equivalent to approximately US $7246 in 2020, based on a mean exchange rate of ¥6.90 per US $1.

### Individual Associations of SDOH and BP Classification With Stroke

During a median follow-up of 5.0 years (IQR, 4.1-5.8 years), 4408 incident stroke events were recorded. Both SDOH and BP classification were independently associated with stroke risk (Table 2). After adjusting for the covariates included in model 2, the results showed that having a high SDOH burden was associated with a significantly increased risk of stroke compared with low SDOH burden (HR, 1.33; 95% CI, 1.23-1.45). In addition, higher BP classification was also associated with increased risk of incident stroke (elevated BP: HR, 1.30 [95% CI, 1.16-1.47]; stage 1 hypertension: HR, 1.53 [95% CI, 1.38-1.69]; and stage 2 hypertension: HR, 1.75 [95% CI, 1.59-1.93]) compared with normal BP. Further adjustment for BP classification resulted in a modest attenuation of the HR for stroke associated with SDOH burden, and adjustment for SDOH burden modestly attenuated the HR for stroke associated with higher BP classification.

**Table 2.** Individual Associations of SDOH Burden and BP Classification With Incident Stroke

SDOH burden and BP classification	Events, No./total No.	Incident rate per 1000 person-years	Model 1^a^		Model 2^b^		Model 3^c^	
			HR (95% CI)	*P* value	HR (95% CI)	*P* value	HR (95% CI)	*P* value
SDOH burden								
Low	671/20 137	6.9	1 [Reference]	NA	1 [Reference]	NA	1 [Reference]	NA
High	3737/70 713	11.0	1.34 (1.24-1.46)	<.001	1.33 (1.23-1.45)	<.001	1.21 (1.10-1.31)	<.001
BP classification								
Normal BP	584/26 834	4.5	1 [Reference]		1 [Reference]		1 [Reference]	
Elevated BP	498/11 772	8.6	1.35 (1.20-1.52)	<.001	1.30 (1.16-1.47)	<.001	1.16 (1.10-1.33)	<.001
Stage 1 hypertension	1208/24 560	10.3	1.64 (1.49-1.81)	<.001	1.53 (1.38-1.69)	<.001	1.40 (1.25-1.56)	<.001
Stage 2 hypertension	2118/27 684	16.3	2.04 (1.86-2.24)	<.001	1.75 (1.59-1.93)	<.001	1.59 (1.42-1.77)	<.001

Abbreviations: BP, blood pressure; HR, hazard ratio; NA, not applicable; SDOH, social determinants of health.

^a^ Model 1 adjusted for age and sex.

^b^ Model 2 additionally adjusted for smoking status, drinking status, body mass index, diabetes, dyslipidemia, and antihypertensive medications.

^c^ Model 3 additionally adjusted for SDOH burden (for the analysis of BP classification) and BP classification (for the analysis of SDOH burden).

### Associations Between BP Classification and Stroke Within SDOH Groups

**Figure 1.**
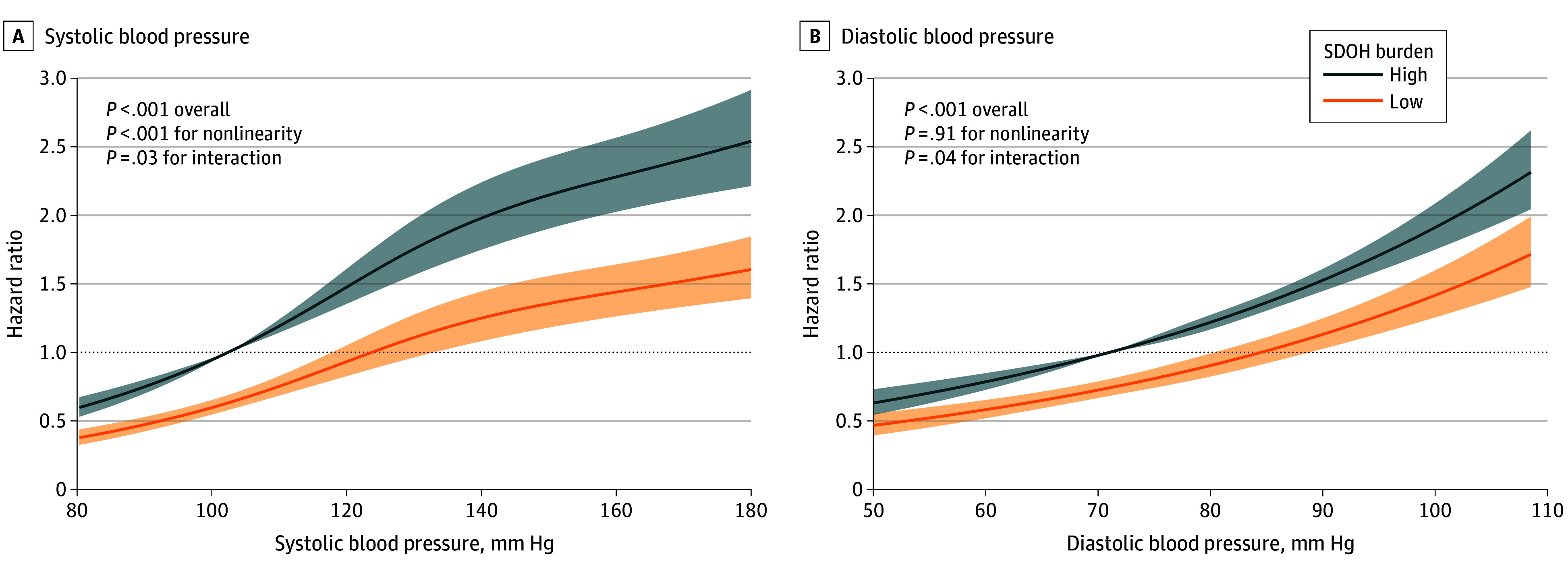
Association of Systolic and Diastolic Blood Pressure With Incident Stroke Stratified by Social Determinants of Health (SDOH) Burden Groups Using Restricted Cubic Spline Models Hazard ratios and 95% CIs were estimated after adjusting for age, sex, smoking status, drinking status, body mass index, diabetes, dyslipidemia, and antihypertensive medications. The restricted cubic spline regression models were conducted with 3 knots at the 25th, 50th, and 75th percentiles of systolic and diastolic blood pressure. Shaded areas indicate 95% CIs.

In the stratified analysis by the level of SDOH burden (Table 3), a significant multiplicative interaction was observed between BP classifications and SDOH burden for stroke risk (*P* = .03 for interaction). Compared with normal BP, elevated BP (HR, 1.33 [95% CI, 1.17-1.52]), stage 1 hypertension (HR, 1.60 [95% CI, 1.43-1.78]), and stage 2 hypertension (HR, 1.79 [95% CI, 1.61-2.00]) were associated with stroke among participants with a high SDOH burden, while only stage 2 hypertension (HR, 1.52 [95% CI, 1.20-1.93]) was associated with stroke among those with a low SDOH burden. Figure 1 illustrates the dose-response associations of SBP and DBP with incident stroke across different SDOH burden groups. The HRs for stroke were higher among participants with a high SDOH burden compared to those with a low burden across the full range of SBP and DBP.

**Table 3.** Association of BP Classification With Incident Stroke Within SDOH Burden Groups

SDOH burden group	Events, No./total No.	Incident rate per 1000 person-years	Model 1^a^	*P* value	*P* value for interaction	Model 2^b^	*P* value	*P* value for interaction
			HR (95% CI)			HR (95% CI)		
Low burden of SDOH					.02			.03
Normal BP	107/6652	3.4	1 [Reference]	NA		1 [Reference]	NA	
Elevated BP	72/2390	6.1	1.21 (0.90-1.63)	.21		1.14 (0.84-1.54)	.40	
Stage 1 hypertension	173/5477	6.5	1.31 (1.03-1.67)	.03		1.20 (0.94-1.54)	.15	
Stage 2 hypertension	319/5618	11.8	1.81 (1.45-2.27)	<.001		1.52 (1.20-1.93)	<.001	
High burden of SDOH								
Normal BP	477/20 182	4.8	1 [Reference]	NA		1 [Reference]	NA	
Elevated BP	426/9382	9.2	1.38 (1.21-1.57)	<.001		1.33 (1.17-1.52)	<.001	
Stage 1 hypertension	1035/19 083	11.4	1.71 (1.54-1.91)	<.001		1.60 (1.43-1.78)	<.001	
Stage 2 hypertension	1799/22 066	17.4	2.09 (1.88-2.31)	<.001		1.79 (1.61-2.00)	<.001	

Abbreviations: BP, blood pressure; HR, hazard ratio; NA, not applicable; SDOH, social determinants of health.

^a^ Model 1 adjusted for age and sex.

^b^ Model 2 additionally adjusted for smoking status, drinking status, body mass index, diabetes, dyslipidemia, and antihypertensive medications.

### Joint Association of SDOH and BP Classification With Stroke

**Figure 2.**
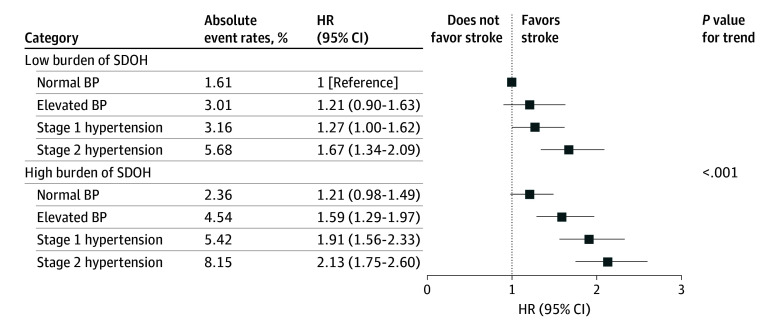
Joint Associations of Social Determinants of Health (SDOH) Burden and Blood Pressure (BP) Classification With Incident Stroke Cox proportional hazards regression models adjusted for age, sex, smoking status, drinking status, body mass index, diabetes, dyslipidemia, and antihypertensive medications. HR indicates hazard ratio.

eTable 4 in the Supplement and Figure 2 show the age- and sex-adjusted and fully adjusted joint associations of SDOH burden and BP classification with stroke, respectively. The cumulative association of both SDOH burden and BP classification with stroke risk appeared in a dose-response pattern. The findings indicated the most substantial risk of stroke among participants with higher BP classifications and concomitant with high SDOH burden (elevated BP: HR, 1.59 [95% CI, 1.29-1.97]; stage 1 hypertension: HR, 1.91 [95% CI, 1.56-2.33]; stage 2 hypertension: HR, 2.13 [95% CI, 1.75-2.60]) compared with those with normal BP and low SDOH burden (Figure 2).

### Stratified and Sensitivity Analyses

Stratified analyses by use of antihypertensive medications revealed similar results for differential associations between BP classification and stroke risk across SDOH burden groups among participants not taking antihypertensive drugs (eTable 5 and eFigure 2 in Supplement 1) and showed similar results for the joint association of BP classification and SDOH with stroke among those not taking antihypertensive drugs (eFigure 3 in Supplement 1). The main results remained robust across various sensitivity analyses. Excluding participants who had a stroke event within 1 year of follow-up (eTable 6 and eFigure 4 in Supplement 1) and using BP classification based on the 2023 ESH guidelines^31^ (eTable 7 and eFigure 5 in Supplement 1) showed similar results.

## Discussion

In this prospective cohort study, we evaluated the risk of stroke associated with BP classification while taking the interaction between BP classification and SDOH burden, as well as their joint association, into account. We observed a significant interaction between BP classification and SDOH burden on stroke risk and found that compared with normal BP, elevated BP, stage 1 hypertension, and stage 2 hypertension were associated with incident stroke among those with high SDOH burden, while only stage 2 hypertension was associated with incident stroke among those with low SDOH burden. We also observed an enhanced stroke risk stratification through joint classification by SDOH burden and BP classification, where the highest risk of stroke was identified among participants with high SDOH burden and stage 2 hypertension.

Previous studies have explored the association between SDOH burden and stroke risk, which were in line with our results.^12,13^ In a prospective cohort study from the Reasons for Geographic and Racial Differences in Stroke study, researchers found that the incidence of stroke was nearly 2 and a half times higher among individuals with high SDOH burden than those with low SDOH burden.^12^ In the current study, we used data from a large-scale, population-based cohort and found similar associations between SDOH burden and stroke among Chinese adults. However, previous studies have focused on the association between stroke and SDOH defined solely by a single dimension, such as the individual level^32,33,34^ or area level^26,35,36^ of SDOH. Our study merged these levels and defined SDOH with 5 factors to assess their combined association with stroke incidence.

The association between BP and the risk of stroke has been widely discussed. An increasing number of individual studies and meta-analyses of observational data have reported that stroke risk begins to increase at the stage of elevated BP, with a progressively higher gradient extending to stage 1 hypertension and stage 2 hypertension.^37,38,39^ A previous meta-analysis indicated that lowering SBP to 130 mm Hg, a threshold designated for stage 1 hypertension, can significantly reduce the risk of stroke.^37^ Similar trends were observed in our study, confirming the association between BP and stroke risk among Chinese adults. However, considering the association of SDOH with BP, we need to delve into more nuanced personalized BP control targets for specific populations. Current evidence indicates that, in the process of stroke risk prevention, there are differences in BP control requirements for populations with different risk factors.^40,41,42^ For instance, BP should be lowered carefully to less than 140/90 mm Hg among patients at high risk for cardiovascular events but without diabetes,^43^ and to less than 130/80 mm Hg among patients with diabetes.^44^ However, currently, many studies that have examined the association between SDOH and BP status mainly use 140/90 mm Hg as the criterion for defining hypertension.^15,19^ Therefore, the BP level associated with increased stroke risk is rarely known for individuals with a high SDOH burden. Our study found that, for populations with a high SDOH burden, the risk of stroke shows a significant upward trend when BP is elevated (defined as SBP of 120-129 mm Hg and DBP of <80 mm Hg), suggesting that a BP of less than 140/90 mm Hg may not meet the stroke prevention needs of populations with a high SDOH burden.

Our study found a significant multiplicative interaction association between BP classification and SDOH burden for the risk of stroke, which emphasizes the importance of BP control in the prevention and control of stroke, especially among individuals with high SDOH burden. However, few studies have investigated the association of the interaction between SDOH burden and BP on the risk of stroke. Although the exact mechanisms behind these associations are largely unclear, there may be several potential explanations for these findings. First, this interaction may be influenced by mental stress in daily life. The high SDOH burden may cause BP to increase by increasing the adaptive load and physiological stress response, ultimately leading to hypertension and triggering stroke.^45^ Second, SDOH factors may shape individuals’ health behaviors, fostering unhealthy practices that are directly associated with stress levels, leading to heightened sympathetic activity, markers of inflammation, and enhanced susceptibility to hypertension and stroke.^46^

### Limitations

Our study has several limitations. First, there is a discrepancy between our definition of SDOH and the definition recommended by the US Department of Health and Human Services’ Healthy People 2030.^28^ This difference may limit the generalizability of our findings to populations assessed using other criteria. Second, SDOH information in our study was collected through self-reported data, which inevitably introduced self-report bias and potential inaccuracies. Third, causal inferences cannot be obtained in an observational study, and the results should be interpreted with caution. Fourth, our follow-up period was relatively short, which may limit the ability to observe long-term outcomes. Future studies with longer follow-up durations are needed to validate our findings.

## Conclusions

Our cohort study demonstrated an elevated stroke risk among participants with higher BP, particularly among those with a high SDOH burden, where even relatively lower hypertension stages have shown increased stroke risk compared with those with low SDOH burden. These findings highlight the need for tailored interventions that effectively lower BP levels and mitigate stroke risk, with a special focus on addressing the unique needs of socioeconomically disadvantaged populations.

## Notes

Supplement 1. **eTable 1.** Baseline Characteristics of Included, Excluded, and SDOH Missing Excluded Populations **eTable 2.** Definitions of Social Determinants of Health and Scoring System **eTable 3.** Baseline Characteristics of Participants According to the 2017 ACC/AHA Guideline BP Classification **eTable 4.** Cross-Classified and Joint Associations of SDOH Burden and BP Classification With Incident Stroke in Age- and Sex-Adjusted Models **eTable 5.** Association of BP Classification With Incident Stroke Across SDOH Burden Groups in Participants Stratified by Taking Antihypertensive Medications **eTable 6.** Association of BP Classification With Incident Stroke Across SDOH Burden Groups After Excluding Stroke Events That Occurred Within the First Year of Follow-Up **eTable 7.** Association of BP Classification Based on the 2023 ESH Guideline With Incident Stroke Across SDOH Burden Groups **eFigure 1.** Flow Chart of Study Participants **eFigure 2.** Association of Systolic and Diastolic Blood Pressure With Incident Stroke Across SDOH Burden Groups in Participants Not Taking (A) and Taking (B) Antihypertensive Medications Using Restricted Cubic Spline Models **eFigure 3.** Joint Associations of SDOH Burden and BP Classification With Incident Stroke in Participants Not Taking (A) and Taking (B) Antihypertensive Medications **eFigure 4.** Joint Associations of SDOH Burden and BP Classification With Incident Stroke After Excluding Stroke Events That Occurred Within the First Year of Follow-Up **eFigure 5.** Joint Associations of SDOH Burden and BP Classification Based on the 2023 ESH Guideline With Incident Stroke

Supplement 2.
Data Sharing Statement
