# Supplementary material for: Social Determinants of Health, Blood Pressure Classification, and Incident Stroke Among Chinese Adults
Source: JAMA Netw Open. 2024 Dec 23;7(12):e2451844. doi: 10.1001/jamanetworkopen.2024.51844 (PMC11667342; doi:10.1001/jamanetworkopen.2024.51844)
Supplement: Supplement 1. — eTable 1. Baseline Characteristics of Included, Excluded, and SDOH Missing Excluded Populations eTable 2. Definitions of Social Determinants of Health and Scoring System eTable 3. Baseline Characteristics of Participants According to the 2017 ACC/AHA Guideline BP Classification eTable 4. Cross-Classified and Joint Associations of SDOH Burden and BP Classification With Incident Stroke in Age- and Sex-Adjusted Models eTable 5. Association of BP Classification With Incident Stroke Across SDOH Burden Groups in Participants Stratified by Taking Antihypertensive Medications eTable 6. Association of BP Classification With Incident Stroke Across SDOH Burden Groups After Excluding Stroke Events That Occurred Within the First Year of Follow-Up eTable 7. Association of BP Classification Based on the 2023 ESH Guideline With Incident Stroke Across SDOH Burden Groups eFigure 1. Flow Chart of Study Participants eFigure 2. Association of Systolic and Diastolic Blood Pressure With Incident Stroke Across SDOH Burden Groups in Participants Not Taking (A) and Taking (B) Antihypertensive Medications Using Restricted Cubic Spline Models eFigure 3. Joint Associations of SDOH Burden and BP Classification With Incident Stroke in Participants Not Taking (A) and Taking (B) Antihypertensive Medications eFigure 4. Joint Associations of SDOH Burden and BP Classification With Incident Stroke After Excluding Stroke Events That Occurred Within the First Year of Follow-Up eFigure 5. Joint Associations of SDOH Burden and BP Classification Based on the 2023 ESH Guideline With Incident Stroke [file jamanetwopen-e2451844-s001.pdf]

Supplementary Online Content

Zhu Y, Wu S, Qiu W, Wang J, Feng Y, Chen C. Social determinants of health, blood pressure classification, and incident stroke among Chinese adults. *JAMA Netw Open.* 2024;7(12):e2451844. doi:10.1001/jamanetworkopen.2024.51844

eTable 1. Baseline Characteristics of Included, Excluded, and SDOH Missing Excluded Populations
eTable 2. Definitions of Social Determinants of Health and Scoring System
eTable 3. Baseline Characteristics of Participants According to the 2017 ACC/AHA Guideline BP Classification
eTable 4. Cross-Classified and Joint Associations of SDOH Burden and BP Classification With Incident Stroke in Age- and Sex-Adjusted Models
eTable 5. Association of BP Classification With Incident Stroke Across SDOH Burden Groups in Participants Stratified by Taking Antihypertensive Medications
eTable 6. Association of BP Classification With Incident Stroke Across SDOH Burden Groups After Excluding Stroke Events That Occurred Within the First Year of Follow-Up
eTable 7. Association of BP Classification Based on the 2023 ESH Guideline With Incident Stroke Across SDOH Burden Groups
eFigure 1. Flow Chart of Study Participants
eFigure 2. Association of Systolic and Diastolic Blood Pressure With Incident Stroke Across SDOH Burden Groups in Participants Not Taking (A) and Taking (B) Antihypertensive Medications Using Restricted Cubic Spline Models
eFigure 3. Joint Associations of SDOH Burden and BP Classification With Incident Stroke in Participants Not Taking (A) and Taking (B) Antihypertensive Medications
eFigure 4. Joint Associations of SDOH Burden and BP Classification With Incident Stroke After Excluding Stroke Events That Occurred Within the First Year of Follow-Up
eFigure 5. Joint Associations of SDOH Burden and BP Classification Based on the 2023 ESH Guideline With Incident Stroke

This supplementary material has been provided by the authors to give readers additional information about their work.

eTable 1. Baseline Characteristics of Included, Excluded, and SDOH Missing Excluded Populations

Characteristics	Included population (N=90,850)	Excluded population (N=11,508)	SDOH Missing Excluded Population (N=6,255)	*P* value [a]	*P* value [b]
Age, median (IQR), y	54.0 (46.0 to 62.0)	55.0 (46.0 to 62.0)	53.0 (45.0 to 63.0)	0.10	0.25
Sex, No. (%)				0.06	0.33
Male	35,460 (39.0)	4,629 (40.2)	2,356 (37.7)		
Female	55,390 (61.0)	6,879 (59.8)	3,899 (62.3)		
BP classification, No. (%)				0.63	0.26
Normal BP	26,834 (29.5)	3,324 (28.9)	1,916 (30.6)		
Elevated BP	11,772 (13.0)	1,545 (13.4)	779 (12.5)		
Stage 1 hypertension	24,560 (27.0)	3,140 (27.3)	1,684 (26.9)		
Stage 2 hypertension	27,684 (30.5)	3,499 (30.4)	1,876 (30.0)		
BMI, median (IQR), kg/m^2	23.9 (21.8 to 26.1)	24.1 (22.0 to 26.3)	23.8 (21.8 to 25.9)	0.23	0.15
Current smoker, No. (%)	15,703 (17.3)	1,966 (17.1)	1,076 (17.2)	0.31	0.87
Current drinker, No. (%)	4,811 (5.3)	622 (5.4)	339 (5.4)	0.89	0.69
Diabetes, No. (%)	13,985 (15.4)	1,771 (15.4)	932 (14.9)	0.83	0.66
Dyslipidemia, No. (%)	16,462 (18.1)	2,138 (18.6)	1,114 (17.8)	0.56	0.55
Antihypertensive medications, No. (%)	16,105 (17.7)	2,099 (18.2)	1,131 (18.1)	0.05	0.49

Abbreviation: SDOH, social determinants of health; IQR, interquartile range; BP, blood pressure; BMI, body mass index.

[a] Excluded population vs. Included population.

[b] SDOH Missing Excluded Population vs. Included population.

© 2024 Zhu Y et al. *JAMA Network Open.*

eTable 2. Definitions of Social Determinants of Health and Scoring System

Components	Questions	Answers	SODH score system
Educational attainment	What is your highest level of education?	1 = Illiteracy<br>2 = Did not complete primary school<br>3 = Primary school<br>4 = Junior high school<br>5 = High school<br>6 = Bachelor's degree<br>7 = Master's degree<br>8 = Doctoral degree<br>9 = Unknown<br>10 = Refused to answer	1 point (high school and above): answer 5, 6, 7, and 8<br>0 point (less than high school): answer 1, 2, 3, and 4<br>Missing: answer 9 and 10
Economic stability	What is your total household income (Yuan) in the past year?	1= 9,999 or less<br>2 = 10,000 ~ 24,999<br>3 = 25,000 ~ 49,999<br>4 = 50,000 ~ 99,999<br>5 = 100,000 ~ 199,999<br>6 = 200,000 ~ 299,999<br>7 = 300,000 ~ 599,999<br>8 = 600,000 or higher<br>9 = Unknown<br>10 = Refused to answer	1 point (50,000 Yuan [a] and above): answer 4, 5, 6, 7, and 8<br>0 point (less than 50,000 Yuan): answer 1, 2, and 3<br>Missing: answer 9 and 10
Health care access	How do you pay for your medical expenses?	1 = Basic Medical Insurance for Urban Residents<br>2 = Basic Medical Insurance for Employees<br>3 = New Rural Cooperative Medical Scheme	1 point (insured): answer 1, 2, 3, and 5<br>0 point (uninsured): answer 5<br>Missing: answer 6 and 7

© 2024 Zhu Y et al. *JAMA Network Open.*

		4 = Commercial Health Insurance<br>5 = No medical insurance or self-paid<br>6 = Unknown<br>7 = Refused to answer	
Social support	What is your marital status?	1 = Married and living with spouse<br>2 = Married and not living with spouse<br>3 = Divorced<br>4 = Widowed<br>5 = Never married<br>6 = Unknown<br>7 = Refused to answer	1 point (married): answer 1 and 2<br>0 point (unmarried): answer 3, 4, and 5<br>Missing: answer 6 and 7
Urban vs. rural residence	Do you live in a rural or urban area?	1 = Urban<br>0 = Rural	1 point (live in urban area): answer 1<br>0 point (live in rural area): answer 0

Abbreviation: SDOH, social determinants of health.

[a] 50,000 Yuan was equivalent to approximately 7,246 USD in 2020, based on an average exchange rate of 6.90 Yuan per 1 USD.

© 2024 Zhu Y et al. *JAMA Network Open.*

eTable 3. Baseline Characteristics of Participants According to the 2017 ACC/AHA Guideline BP Classification

**Characteristics**	**Total population (N=90,850)**	**Normal BP (N=26,834)**	**Elevated BP (N=11,772)**	**Stage 1 hypertension (N=24,560)**	**Stage 2 hypertension (N=27,684)**	***P* value**
Age, median (IQR), y	54.0 (46.0 to 62.0)	48.0 (42.0 to 56.0)	54.0 (46.0 to 62.0)	54.0 (46.0 to 62.0)	58.0 (50.0 to 65.0)	< 0.001
Sex, No. (%)						< 0.001
Male	35,460 (39.0)	8,404 (31.3)	4,401 (37.4)	10,854 (44.2)	11,801 (42.6)	
Female	55,390 (61.0)	18,430 (68.7)	7,371 (62.6)	13,706 (55.8)	15,883 (57.4)	
Education, No. (%)						< 0.001
Less than high school	87,927 (96.8)	25,469 (94.9)	11,425 (97.1)	23,859 (97.1)	27,174 (98.2)	
High school or above	2,923 (3.2)	1,365 (5.1)	347 (2.9)	701 (2.9)	510 (1.8)	
Household income, No. (%)						< 0.001
Less than 50,000 Yuan [a]	47,191 (51.9)	12,882 (48.0)	6,019 (51.1)	12,817 (52.2)	15,473 (55.9)	
50,000 Yuan or above	43,659 (48.1)	13,952 (52.0)	5753 (48.9)	11,743 (47.8)	12,211 (44.1)	
Health insurance, No. (%)						< 0.001
Uninsured	3,964 (4.4)	1,246 (4.6)	432 (3.7)	1,076 (4.4)	1,210 (4.4)	
Insured	86,886 (95.6)	25,588 (95.4)	11,340 (96.3)	23,484 (95.6)	26,474 (95.6)	
Social support, No. (%)						< 0.001
Unmarried	5,004 (5.5)	1,330 (5.0)	647 (5.5)	1,260 (5.1)	1,767 (6.4)	
Married	85,846 (94.5)	25,504 (95.0)	11,125 (94.5)	23,300 (94.9)	25,917 (93.6)	
Urban vs. rural residence, No. (%)						< 0.001
Rural	48,187 (53.0)	14,231 (53.0)	6,828 (58.0)	12,786 (52.1)	14,342 (51.8)	
Urban	42,663 (47.0)	12,603 (47.0)	4,944 (42.0)	11,774 (47.9)	13,342 (48.2)	
SDOH burden, No. (%)						< 0.001
Low burden of SDOH	20,137 (22.2)	6,652 (24.8)	2,390 (20.3)	5,477 (22.3)	5,618 (20.3)	
High burden of SDOH	70,713 (77.8)	20,182 (75.2)	9,382 (79.7)	19,083 (77.7)	22,066 (79.7)	

© 2024 Zhu Y et al. *JAMA Network Open.*

Antihypertensive medications, No. (%)	16,105 (17.7)	972 (3.6)	1,191 (10.1)	4,090 (16.7)	9,852 (35.6)	< 0.001
BMI, median (IQR), kg/m^2	23.9 (21.8 to 26.1)	22.8 (20.9 to 24.8)	23.7 (21.6 to 25.9)	24.2 (22.2 to 26.4)	24.8 (22.7 to 27.1)	< 0.001
Current smoker, No. (%)	15,703 (17.3)	4,135 (15.4)	1,933 (16.4)	4,621 (18.8)	5,014 (18.1)	< 0.001
Current drinker, No. (%)	4,811 (5.3)	1,024 (3.8)	483 (4.1)	1,393 (5.7)	1,911 (6.9)	< 0.001
Diabetes, No. (%)	13,985 (15.4)	2,358 (8.8)	1,664 (14.1)	3,954 (16.1)	6,009 (21.7)	< 0.001
Dyslipidemia, No. (%)	16,462 (18.1)	3,642 (13.6)	2,213 (18.8)	4,517 (18.4)	6,090 (22.0)	< 0.001

Abbreviation: ACC, American College of Cardiology; AHA, American Heart Association; BP, blood pressure; IQR, interquartile range; SDOH, social determinants of health; BMI, body mass index.

[a] 50,000 Yuan was equivalent to approximately 7,246 USD in 2020, based on an average exchange rate of 6.90 Yuan per 1 USD.

© 2024 Zhu Y et al. *JAMA Network Open.*

eTable 4. Cross-Classified and Joint Associations of SDOH Burden and BP Classification With Incident Stroke in Age- and Sex-Adjusted Models

	Absolute event rates, %	Model 1 [a]	
		HR (95%CI)	*P* value
**Low burden of SODH**			
Normal BP	1.61	1 [Reference]	
Elevated BP	3.01	1.26 (0.93-1.70)	0.13
Stage 1 hypertension	3.16	1.36 (1.07-1.73)	0.01
Stage 2 hypertension	5.68	1.93 (1.55-2.40)	< 0.001
**High burden of SODH**			
Normal BP	2.36	1.21 (0.98-1.49)	0.08
Elevated BP	4.54	1.65 (1.33-2.04)	< 0.001
Stage 1 hypertension	5.42	2.06 (1.69-2.51)	< 0.001
Stage 2 hypertension	8.15	2.49 (2.05-3.03)	< 0.001

[a] Model 1 adjusted for age and sex.

Abbreviation: SDOH, social determinants of health; BP, blood pressure; HR, hazard ratio; CI, confidence interval.

© 2024 Zhu Y et al. *JAMA Network Open.*

eTable 5. Association of BP Classification With Incident Stroke Across SDOH Burden Groups in Participants Stratified by Taking Antihypertensive Medications

	**Model 1 [a]**			**Model 2 [b]**		
	**HR (95% CI)**	***P* value**	***P* for interaction**	**HR (95% CI)**	***P* value**	***P* for interaction**
**Not taking antihypertensive medications**						
**Low burden of SODH**						
Normal BP	1 [Reference]			1 [Reference]		
Elevated BP	1.19 (0.85-1.66)	0.32		1.15 (0.82-1.62)	0.41	
Stage 1 hypertension	1.27 (0.96-1.67)	0.09		1.23 (0.93-1.62)	0.14	
Stage 2 hypertension	1.67 (1.29-2.17)	< 0.001	0.03	1.57 (1.20-2.05)	0.001	0.03
**High burden of SODH**						
Normal BP	1 [Reference]			1 [Reference]		
Elevated BP	1.29 (1.11-1.49)	< 0.001		1.28 (1.11-1.48)	< 0.001	
Stage 1 hypertension	1.62 (1.44-1.83)	< 0.001		1.61 (1.43-1.82)	< 0.001	
Stage 2 hypertension	1.80 (1.60-2.03)	< 0.001		1.78 (1.58-2.00)	< 0.001	
**Taking antihypertensive medications**						
**Low burden of SODH**						
Normal BP	1 [Reference]			1 [Reference]		
Elevated BP	0.85 (0.43-1.69)	0.65		0.84 (0.42-1.66)	0.61	
Stage 1 hypertension	0.81 (0.47-1.42)	0.47		0.83 (0.47-1.45)	0.50	
Stage 2 hypertension	1.04 (0.62-1.75)	0.87	0.04	1.03 (0.62-1.74)	0.90	0.04
**High burden of SODH**						
Normal BP	1 [Reference]			1 [Reference]		
Elevated BP	1.32 (0.94-1.84)	0.11		1.31 (0.94-1.83)	0.11	
Stage 1 hypertension	1.33 (1.00-1.79)	0.05		1.34 (1.00-1.79)	0.05	
Stage 2 hypertension	1.54 (1.17-2.03)	0.002		1.56 (1.18-2.06)	0.002	

Abbreviation: BP, blood pressure; SDOH, social determinants of health; HR, hazard ratio; CI, confidence interval.

[a] Model 1 adjusted for age and sex.

[b] Model 2 additionally adjusted for smoking status, drinking status, body mass index, diabetes, and dyslipidemia.

© 2024 Zhu Y et al. *JAMA Network Open.*

eTable 6. Association of BP Classification With Incident Stroke Across SDOH Burden Groups After Excluding Stroke Events That Occurred Within the First Year of Follow-Up

	Events/N	Incident rate per 1,000 person-years	Model 1 [a]			Model 2 [b]		
			HR (95% CI)	*P* value	*P* for interaction	HR (95% CI)	*P* value	*P* for interaction
**Low burden of SODH**								
Normal BP	88/6633	2.76	1 [Reference]			1 [Reference]		
Elevated BP	61/2379	5.20	1.24 (0.89-1.72)	0.20		1.16 (0.83-1.61)	0.39	
Stage 1 hypertension	146/5450	5.50	1.34 (1.03-1.75)	0.03		1.21 (0.93-1.59)	0.16	
Stage 2 hypertension	257/5556	9.50	1.77 (1.38-2.27)	< 0.001	0.03	1.46 (1.13-1.89)	0.004	0.04
**High burden of SODH**								
Normal BP	393/20098	3.97	1 [Reference]			1 [Reference]		
Elevated BP	357/9313	7.74	1.39 (1.21-1.61)	< 0.001		1.35 (1.17-1.56)	< 0.001	
Stage 1 hypertension	839/18887	9.24	1.7 (1.51-1.92)	< 0.001		1.59 (1.4-1.79)	< 0.001	
Stage 2 hypertension	1417/21684	13.76	2.01 (1.8-2.26)	< 0.001		1.74 (1.54-1.96)	< 0.001	

Abbreviation: BP, blood pressure; SDOH, social determinants of health; HR, hazard ratio; CI, confidence interval.

[a] Model 1 adjusted for age and sex.

[b] Model 2 additionally adjusted for smoking status, drinking status, body mass index, diabetes, dyslipidemia, and antihypertensive medications.

© 2024 Zhu Y et al. *JAMA Network Open.*

eTable 7. Association of BP Classification Based on the 2023 ESH Guideline * With Incident Stroke Across SDOH Burden Groups

	Events/N	Incident rate per 1,000 person-years	Model 1 [a]			Model 2 [b]		
			HR (95% CI)	*P* value	*P* for interaction	HR (95% CI)	*P* value	*P* for interaction
**Low burden of SODH**								
Optimal BP	91/6,428	2.95	Reference		0.02	Reference		0.01
Normal BP	10/896	2.30	0.70 (0.37-1.35)	0.29		0.69 (0.36-1.32)	0.26	
High-normal BP	131/3,920	6.80	1.50 (1.14-1.96)	0.003		1.44 (1.09-1.89)	0.009	
Hypertension	439/8,893	10.26	1.84 (1.46-2.32)	< 0.001		1.71 (1.35-2.16)	< 0.001	
**High burden of SODH**								
Optimal BP	424/19,434	4.44	Reference			Reference		
Normal BP	110/2,965	7.75	1.57 (1.27-1.93)	< 0.001		1.56 (1.26-1.93)	< 0.001	
High-normal BP	744/14,161	10.93	1.69 (1.50-1.91)	< 0.001		1.66 (1.47-1.87)	< 0.001	
Hypertension	2,459/34,153	15.22	2.03 (1.83-2.26)	< 0.001		1.95 (1.75-2.17)	< 0.001	

Abbreviation: BP, blood pressure; ESH, European Society of Hypertension; SDOH, social determinants of health; HR, hazard ratio; CI, confidence interval.

[*] BP classifications based on the 2023 ESH Guideline include optional BP (SBP < 120 mmHg and DBP < 80 mmHg), normal BP (SBP 120-129 mm Hg and DBP 80-84 mm Hg), high-normal BP (SBP 130-139 mm Hg and/or DBP 85-89 mm Hg), and hypertension (SBP ≥ 140 mm Hg and/or DBP ≥ 90 mm Hg).

[a] Model 1 adjusted for age and sex.

[b] Model 2 additionally adjusted for smoking status, drinking status, body mass index, diabetes, dyslipidemia, and antihypertensive medications.

© 2024 Zhu Y et al. *JAMA Network Open.*

eFigure 1. Flow Chart of Study Participants

SDOH indicates social determinants of health; CHD, coronary heart disease; HF, heart failure.

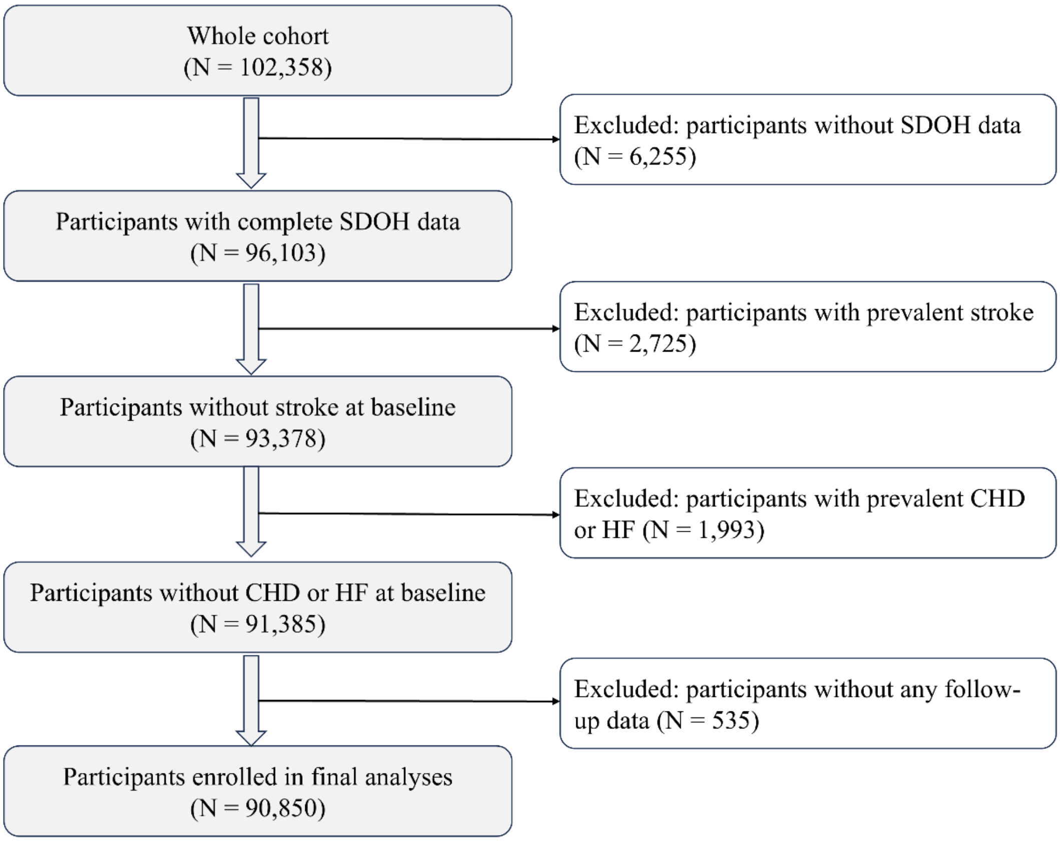

© 2024 Zhu Y et al. *JAMA Network Open.*

eFigure 2. Association of Systolic and Diastolic Blood Pressure With Incident Stroke Across SDOH Burden Groups in Participants Not Taking (A) and Taking (B) Antihypertensive Medications Using Restricted Cubic Spline Models

Hazard ratios (solid lines) and 95% CIs (shaded area) were estimated after adjusting for age, sex, smoking status, drinking status, body mass index, diabetes, and dyslipidemia. The restricted cubic spline regression models were conducted with 3 knots at the 25th, 50th, and 75th percentiles of systolic and diastolic blood pressure.

SDOH indicates social determinants of health; CI, confidence interval; SBP, systolic blood pressure; DBP, diastolic blood pressure.

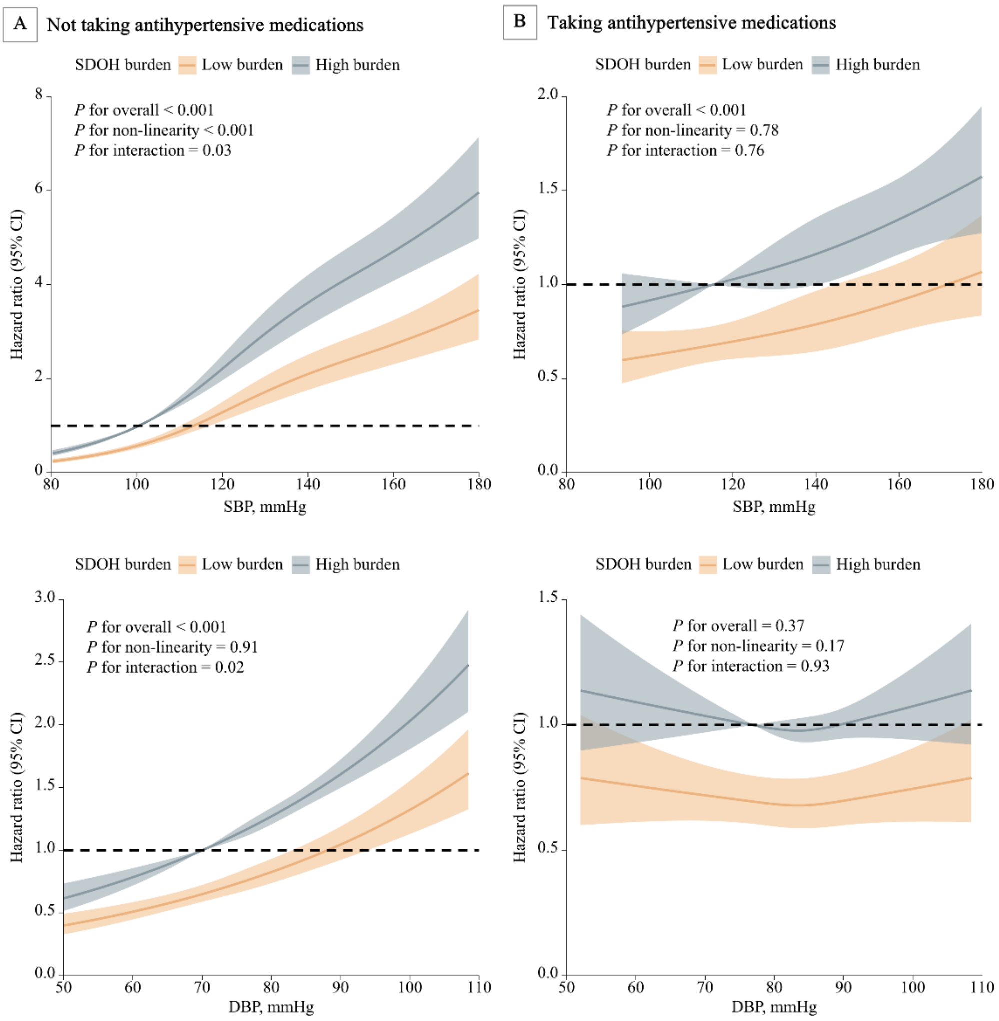

© 2024 Zhu Y et al. *JAMA Network Open.*

eFigure 3. Joint Associations of SDOH Burden and BP Classification With Incident Stroke in Participants Not Taking (A) and Taking (B) Antihypertensive Medications

Cox regression models adjusted for age, sex, smoking status, drinking status, body mass index, diabetes, and dyslipidemia.

SDOH indicates social determinants of health; BP, blood pressure; HR, hazard ratio; CI, confidence interval.

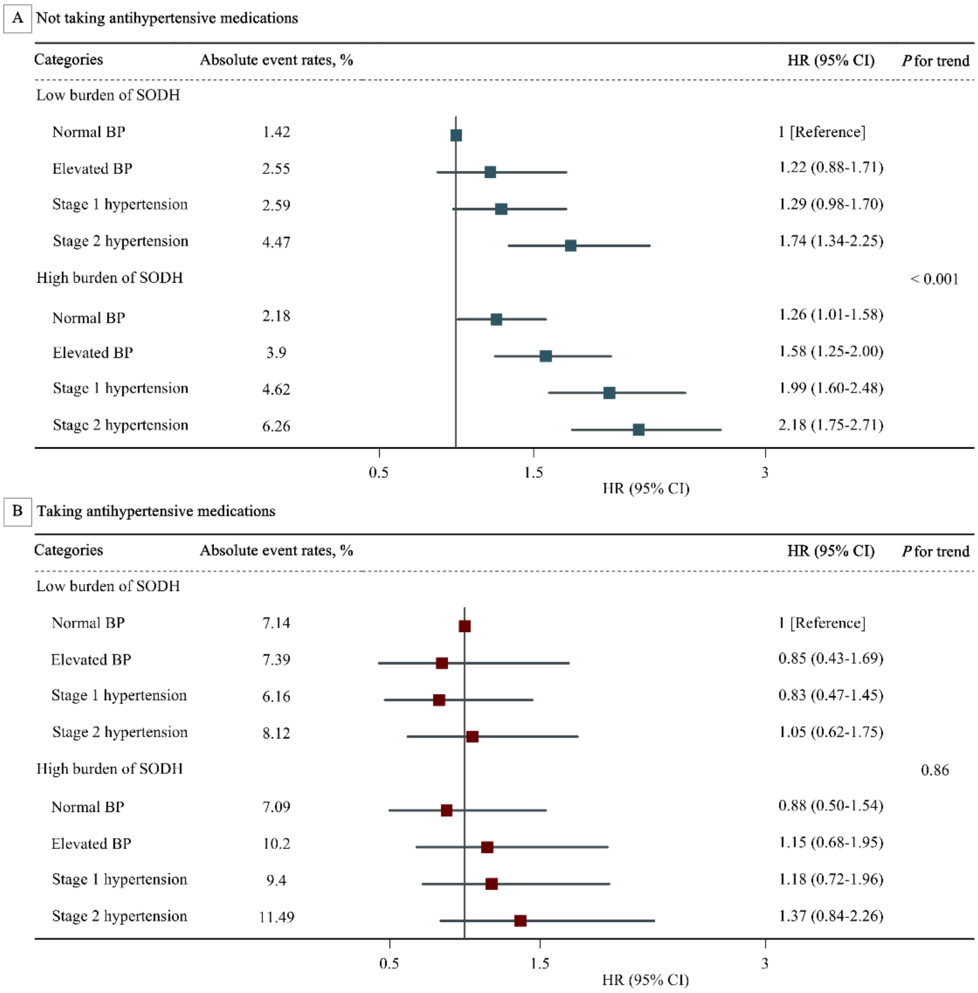

© 2024 Zhu Y et al. *JAMA Network Open.*

eFigure 4. Joint Associations of SDOH Burden and BP Classification With Incident Stroke After Excluding Stroke Events That Occurred Within the First Year of Follow-Up

Cox regression models adjusted for age, sex, smoking status, drinking status, body mass index, diabetes, dyslipidemia, and antihypertensive medications.

SDOH indicates social determinants of health; BP, blood pressure; HR, hazard ratio; CI, confidence interval.

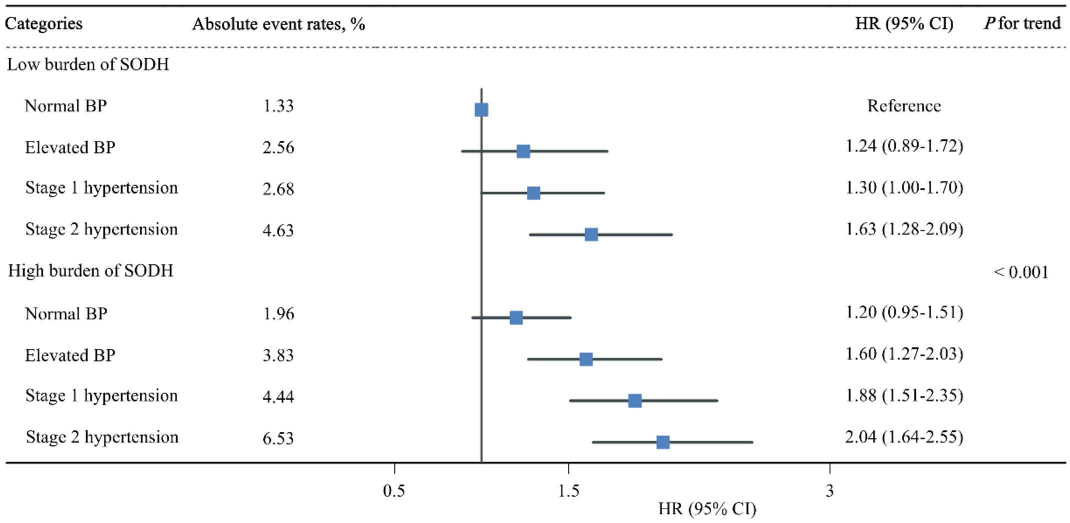

© 2024 Zhu Y et al. *JAMA Network Open.*

eFigure 5. Joint Associations of SDOH Burden and BP Classification Based on the 2023 ESH Guideline [*] With Incident Stroke

[*] BP classifications based on the 2023 ESH Guideline include optional BP (SBP < 120 mmHg and DBP < 80 mmHg), normal BP (SBP 120-129 mm Hg and DBP 80-84 mm Hg), high-normal BP (SBP 130-139 mm Hg and/or DBP 85-89 mm Hg), and hypertension (SBP ≥ 140 mm Hg and/or DBP ≥ 90 mm Hg).

Cox regression models adjusted for age, sex, smoking status, drinking status, body mass index, diabetes, dyslipidemia, and antihypertensive medications.

SDOH indicates social determinants of health; BP, blood pressure; ESH, European Society of Hypertension; HR, hazard ratio; CI, confidence interval.

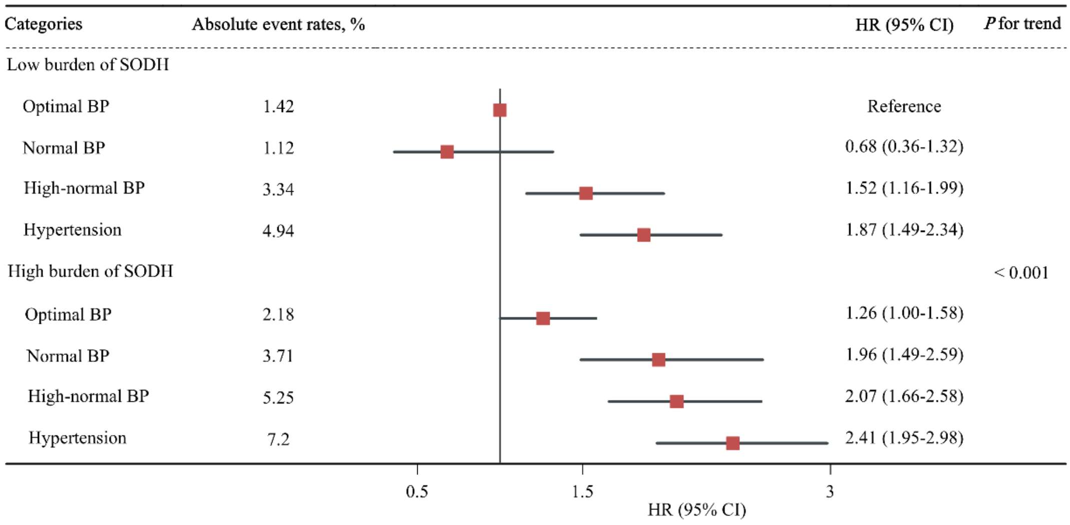

© 2024 Zhu Y et al. *JAMA Network Open.*
