# Supplementary material for: Social Determinants of Health, Blood Pressure Classification, and Incident Stroke Among Chinese Adults
Source: JAMA Netw Open. 2024 Dec 23;7(12):e2451844. doi: 10.1001/jamanetworkopen.2024.51844 (PMC11667342; doi:10.1001/jamanetworkopen.2024.51844)
Supplement: Supplement 2. — Data Sharing Statement [file jamanetwopen-e2451844-s002.pdf]

Data Sharing Statement

Zhu. Social Determinants of Health, Blood Pressure Classification, and Incident Stroke Among Chinese Adults. *JAMA Netw Open*. Published December 23, 2024.
doi:10.1001/jamanetworkopen.2024.51844

Data
Data available: No

Additional Information
Explanation for why data not available: The underlying data can be shared on reasonable request to the corresponding author.
